# Global Geographic Diversity and Distribution of the Myxobacteria

**DOI:** 10.1128/spectrum.00012-21

**Published:** 2021-07-14

**Authors:** Jingjing Wang, Jianing Wang, Shuge Wu, Zheng Zhang, Yuezhong Li

**Affiliations:** a State Key Laboratory of Microbial Technology, Institute of Microbial Technology, Shandong University, Qingdao, People’s Republic of China; b Suzhou Research Institute, Shandong University, Suzhou, People’s Republic of China; Howard University

**Keywords:** *Myxococcales*, geographical diversity, global distribution, cultivation proportion, genome-sequenced proportion, Earth Microbiome Project data

## Abstract

Bacteria are globally distributed in various environments on earth, but a global view of the geographic diversity and distribution of a single taxon is lacking. The Earth Microbiome Project (EMP) has established a global collection of microbial communities, providing the possibility for such a survey. *Myxococcales* is a bacterial order with a potent ability to produce diverse natural products and have wide application potential in agriculture, biomedicine, and environmental protection. In this study, through a comparative analysis of the EMP data and public information, we determined that myxobacteria account for 2.34% of the total bacterial operational taxonomic units (OTUs), and are one of the most diverse bacterial groups on Earth. *Myxococcales* OTUs are globally distributed and prefer nonsaline soil and sediments, followed by saline environments, but rarely appear in host-associated environments. Myxobacteria are among the least-investigated bacterial groups. The presently cultured and genome-sequenced myxobacteria are most likely environmentally widespread and abundant taxa, and account for approximately 10% and 7% of the myxobacterial community (>97% similarity), respectively. This global panoramic view of the geographic distribution and diversity of myxobacteria, as well as their cultured and genome-sequenced information, will enable us to explore these important bioresources more reasonably and efficiently. The diversity and distribution of myxobacteria beyond the EMP data are further discussed.

**IMPORTANCE** The diversity and distribution of bacteria are crucial for our understanding of their ecological importance and application potential. Myxobacteria are fascinating prokaryotes with multicellular behaviors and a potent capacity for producing secondary metabolites, and have a wide range of potential applications. The ecological importance of myxobacteria in major ecosystems is becoming established, but the global geographic diversity and distribution remain unclear. From a global survey we revealed that *Myxococcales* OTUs are globally distributed and prefer nonsaline soil and sediments, followed by saline environments, but rarely appear in host-associated environments. The global panoramic view of the geographic distribution and diversity of myxobacteria, as well as their cultured and genome-sequenced information, will enable us to explore these important bioresources more reasonably and efficiently.

## INTRODUCTION

Bacteria are the most widespread form of life on Earth (1, 2). Arduous isolation work of bacterial strains is the premise for research and development of their metabolic capabilities in industry and medicine, and requires a global view of their geographic diversity and distribution, which, however, is lacking. The Gram-negative gliding myxobacteria are characterized by their sophisticated multicellular lifestyle (3) and have wide application potential in agriculture, biomedicine, and environmental protection. For example, myxobacteria are able to produce a variety of bioactive secondary metabolites and are one of the most important bacterial resources for the discovery of novel antibiotics; more than 100 new carbon skeleton metabolites and over 600 derivatives have been identified from myxobacterial strains (4, 5). Some myxobacteria produce diverse carotenoids (6), degrade 2-chlorophenol (7), and reduce uranium (VI) (8). In addition, myxobacteria are able to prey on many other bacteria and fungi, and are micropredators that regulate bacterial communities in agricultural land (9). Some myxobacteria can prevent and control cucumber Fusarium wilt by regulating the soil microbial community (10).

Although presently regarded as a phylum (*Myxococcota*), according to the genome analysis and functional capabilities (11), myxobacteria are phylogenetically classified into an order (*Myxococcales*) of Deltaproteobacteria based on their 16S rRNA gene sequences (12). Based on the cultured myxobacteria, the *Myxococcales* order is divided into three identified suborders, i.e., *Cystobacterineae*, *Sorangiineae*, and *Nannocystineae*. Because of their complex social characteristics, myxobacteria have been regarded as typical soil dwellers for a long time, and cultured myxobacteria are typically obtained from terrestrial environments such as neutral or slightly alkaline soil, decaying plant materials, rotting wood, the bark of dead or living trees, and the dung of herbivores (13, 14). In addition, myxobacteria have also been isolated in some extreme conditions, such as marine conditions (15), saline-alkaline soils (16), arid deserts (17), hot springs (18), and Antarctic soils (19). Moreover, culture-independent high-throughput sequencing has revealed that myxobacteria are more diverse than was thought and that myxobacterial sequences are ubiquitous and fairly predominant not only in soils (20–23) but also in various marine sediments (24, 25) or limnetic sediments (26). The myxobacterial communities have also been investigated in other environments, including acidic high moors and fens (27), island sand and compost (28), and saprolite subsurface environments (29). Myxobacteria were even detected in the deepest ocean on Earth, Challenger Deep, with a water depth of 11 km (30). Some specific myxobacterial taxa were found to be present in high abundance in activated sludge habitat (31, 32). Recently, a meta-16S rRNA gene phylogenetic reconstruction suggested that *Myxococcales* could be divided into 20 suborders, 58 families, 445 genera, and 998 species (33). However, although myxobacteria are found in a wide variety of habitats and their ecological importance in major ecosystems is becoming established, the global distribution and geographic diversity information of myxobacteria across different environments still remains unclear.

The Earth Microbiome Project (EMP) was founded in 2010 to sample the Earth’s microbial communities at an unprecedented scale (1, 34, 35). In this study, based on EMP data and public information on myxobacteria, we analyzed myxobacterial diversity and distributions in different environments at the global scale. The aim of this study was (i) to evaluate the diversity of the myxobacterial community on Earth, (ii) to depict the global geographic distribution of myxobacteria in different types of environment, and (iii) to calculate the proportions of currently cultured and genome-sequenced myxobacteria. This was the first study to evaluate the global geographic distribution of a specific bacterial taxonomic group. The results described in this paper will provide a panoramic view of myxobacteria on Earth, and the information will help us understand the natural and ecological characteristics of myxobacteria and guide our exploration and application of these important resources.

## RESULTS

### Myxobacteria are one of the most diverse and globally distributed bacteria, with a preference for nonsaline soil environments.

**FIG 1:**
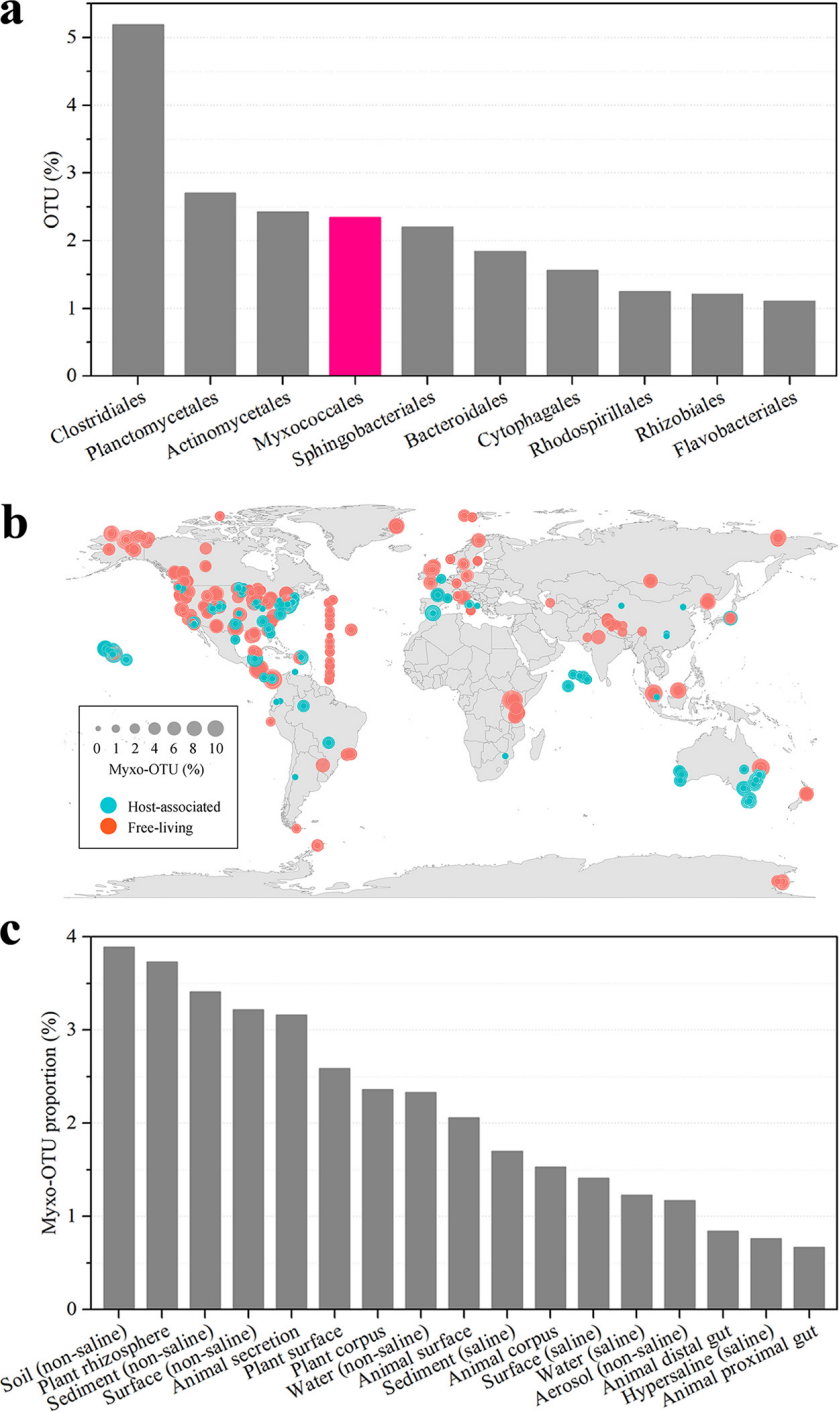

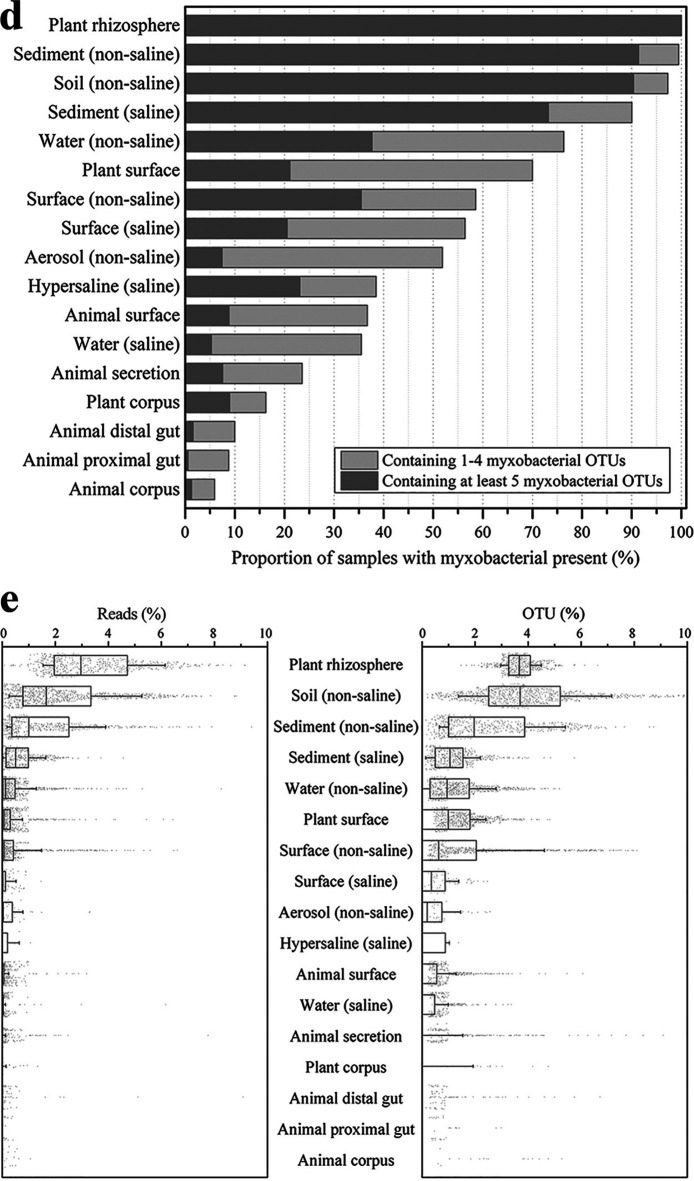
Diversity and distribution of myxobacteria on Earth based on 10,000 EMP samples. (a) The top 10 orders ranked by OTU number in the prokaryotes revealed from the EMP data. (b) Global geographic diversity and distribution of the myxobacteria community. (c) Proportions of myxobacterial OTUs in the 17 environment types. (d) Proportion of the samples containing myxobacterial OTUs in samples from the 17 environment types. The environment types were classified by EMPO. (e) Proportions of myxobacterial reads and taxa in the prokaryotic biomes of the 17 environment types. Each gray point represents a single sample. For the box plots, the middle line indicates the median, the box represents the 25th to 75th percentiles, and the error bar indicates the 10th to 90th percentiles of observations.

A 10,000-sample subset of the EMP data was considered representative for different environment types and studies (1). We employed a 10,000-sample subset (5,000 sequences were randomly selected from each sample) to evaluate the distribution and diversity of the myxobacterial community on Earth. The meta-analysis of the 50,000,000 16S rRNA gene sequences revealed 262,011 operational taxonomic units (OTUs), and 35% of them were identified into 144 known prokaryotic orders (at the 70% default confidence threshold) by the Ribosomal Database Project (RDP) classifier (36). In total, 6,133 OTUs were identified in the *Myxococcales* order (Table S1 in the supplemental material), which accounted for 2.34% of the total OTUs. *Myxococcales* ranked 4th following the orders of *Clostridiales* (5.19%), *Planctomycetales* (2.70%), and *Actinomycetales* (2.43%) (Fig. 1a). The results indicated that *Myxococcales* is one of the most diverse bacteria on Earth. However, the relative abundance of *Myxococcales* (0.67%) only ranked 25th among the prokaryotic orders revealed from the EMP data at the global scale (see Fig. S1).

The EMP ontology (EMPO) classified the environment samples at three levels: level 1 was divided into free-living or host-associated; level 2 was divided into saline or nonsaline (if free-living) and animal or plant (if host-associated); and level 3 contained 17 types (1). As shown in Fig. 1b, myxobacterial OTUs were distributed worldwide, including free-living samples and host-associated samples. The myxobacterial OTUs were found in each of the 17 environment types, and exceeded 1% of the total OTU numbers in 14 environment types (Fig. 1c). The myxobacterial OTU diversity was in the top 5 among the orders in 8 environment types, including different nonsaline samples (except aerosols), saline sediments, plant rhizospheres and surfaces, and animal secretions (Table S2 summarizes the proportion of myxobacterial OTUs and their ranks in different environment types). Our results indicated that *Myxococcales* is among the most diverse and widely distributed prokaryotic orders on Earth, not only in nonsaline soils but also in many other environments.

The distribution of myxobacteria varied in different environment types (Fig. 1d). For example, myxobacterial OTUs were detected in almost every sample from the plant rhizosphere, nonsaline sediments, and soils. In the saline sediments, 90% of the samples contained myxobacterial OTUs. In contrast, the myxobacterial OTUs appeared in less than 10% of the samples from animal corpus, proximal gut, or distal gut, which suggested the appearance in these samples was somewhat incidental. To reduce accidental interference, we further analyzed the proportion of the samples containing at least 5 myxobacterial OTUs. The results showed that more than 90% of the samples from the nonsaline plant rhizosphere, sediment, and soil contained myxobacteria, and the corresponding value also exceeded 70% of the saline sediment samples (Fig. 1d). In contrast, almost all the samples from the animal corpus, proximal gut, and distal gut did not possess more than 5 myxobacterial OTUs, and less than 10% of samples from the plant corpus, animal secretion and surface, saline water, and nonsaline aerosol had at least 5 myxobacterial OTUs. A total of 20% to 40% of the samples from nonsaline water, plant surface, nonsaline or saline surface and hypersaline water contained at least 5 myxobacterial OTUs. Accordingly, the plant rhizosphere, soil (nonsaline), sediment (nonsaline), and sediment (saline) were the major habitats possessing diverse myxobacteria. In addition, myxobacteria often appeared in the environmental samples of water (nonsaline), plant surface, surface (nonsaline or saline) and hypersaline (saline). In other environment types, such as animal-associated environments, myxobacteria are normally rare, suggesting that these environments are not suitable habitats for myxobacterial survival.

We further calculated the proportion of myxobacterial reads and taxa in the prokaryotic biomes (Fig. 1e; the sample number for each of the 17 environment types refers to Table S1). For the 552 plant rhizosphere samples, the median proportion of myxobacterial reads in a single sample was 2.96% and the upper and lower quartiles were 4.72% and 1.96%, respectively. For the 954 nonsaline soil samples, the median proportion of myxobacterial reads also reached 1.66% (interquartile range [IQR] 0.78% to 3.34%). The corresponding values of the 544 nonsaline sediment samples, 541 saline sediment samples, and 954 nonsaline water samples were 1.00% (IQR 0.36% to 2.51%), 0.50% (IQR 0.14% to 0.98%), and 0.12% (IQR 0.02% to 0.48%), respectively. Notably, compared to the read proportion, the median myxobacterial OTU proportion reached 3.70% (IQR 2.51% to 5.19%) in the nonsaline soil samples; 3.66% (IQR 3.27% to 4.08%) in the plant rhizosphere samples; and 1.96% (IQR 1.00% to 3.86%), 1.05% (IQR 0.50% to 1.54%), and 0.94% (IQR 0.30% to 1.77%) in the sediment (nonsaline), sediment (saline), and water (nonsaline) samples, respectively. The myxobacterial OTU proportions were higher than the read proportions; this result suggested that the diversity of myxobacteria was normally higher than the average diversity of prokaryotes in different environments.

### Different myxobacterial taxa have preferred environments.

**FIG 2:**
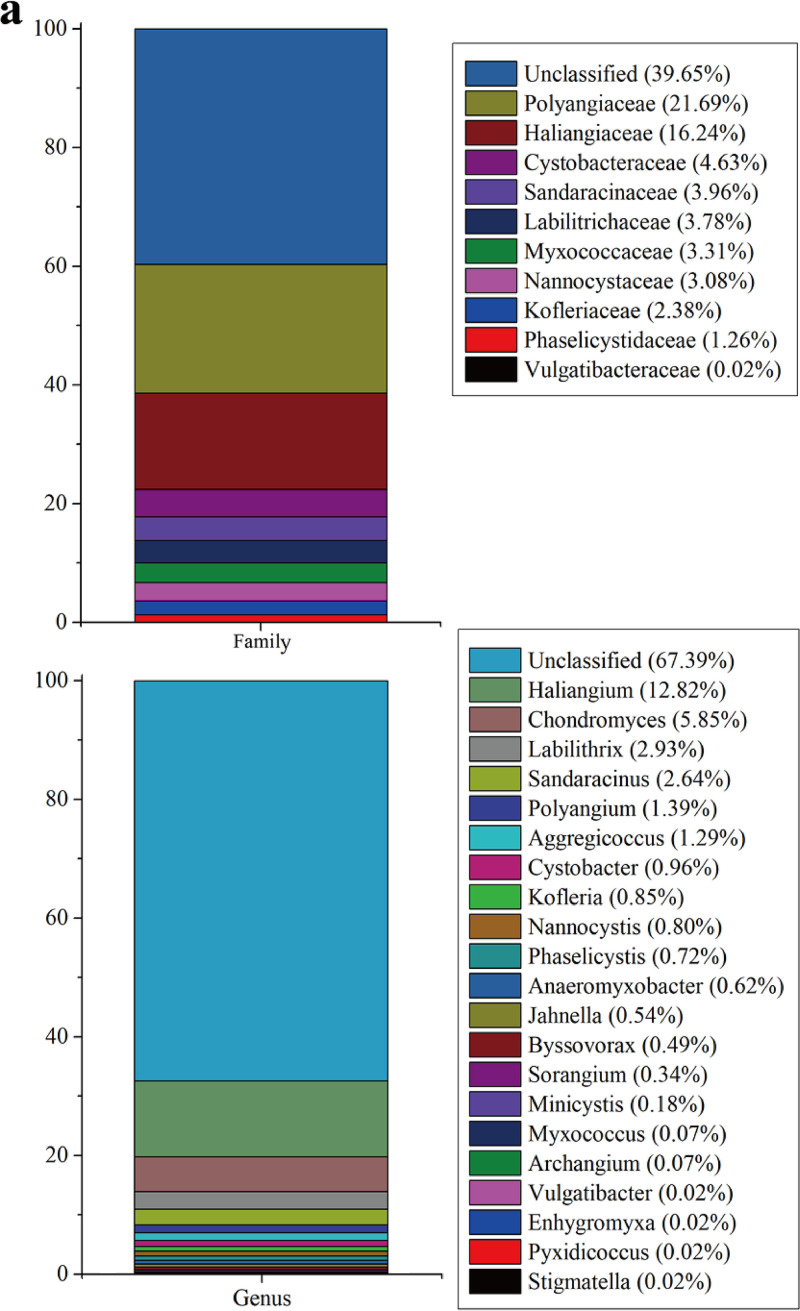

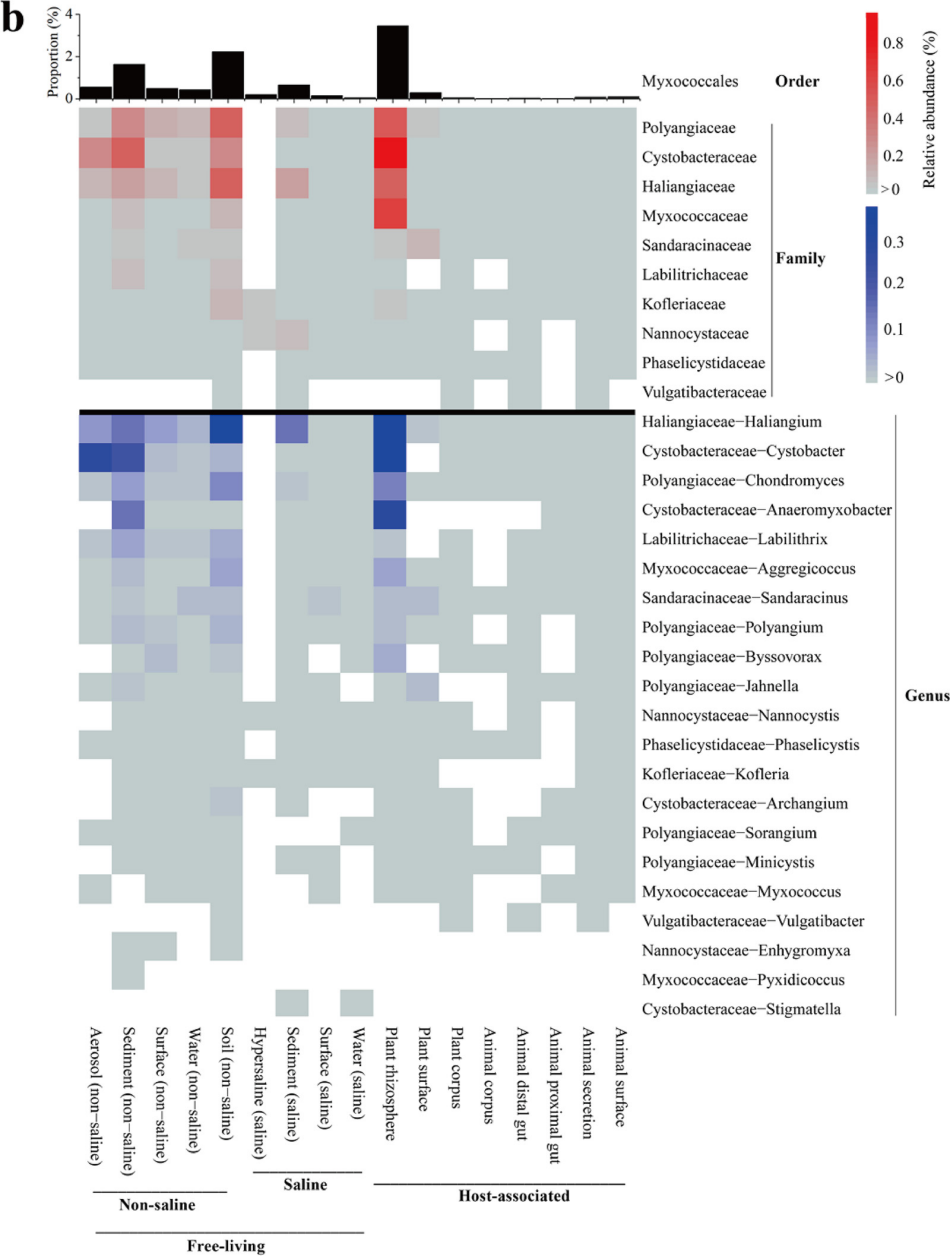
Proportion and composition of the *Myxococcales* order in 17 environment types. (a) The identified families and genera of myxobacteria. (b) Heatmap of myxobacterial relative abundance in 17 environment types at the family level or the genus level.

Based on the RDP classification, 60.35% and 32.61% of the myxobacterial OTUs identified in the EMP data were classified into the known families and genera of *Myxococcales*, respectively (Fig. 2a). At the family level, except for *Vulgatibacteraceae*, of which only one OTU (>97% similarity) was identified, the other nine myxobacterial families contained at least 77 OTUs (Table S1). The most diverse families, *Polyangiaceae* and *Haliangiaceae*, contained 1,330 and 996 OTUs, respectively, ranking 12th and 16th among the 354 prokaryotic families identified in the EMP data. The OTU proportions affiliated with *Polyangiaceae* and *Haliangiaceae* accounted for 21.69% and 16.24% of the total myxobacterial OTUs, respectively. Comparably, the proportions of any of the other 8 families did not exceed 5%. At the genus level, the most abundant genera, *Haliangium* and *Chondromyces*, contained 786 and 359 OTUs, respectively, ranking 10th and 35th out of the total 1,698 prokaryotic genera identified in the EMP data, respectively. The OTUs of *Haliangium* and *Chondromyces* accounted for 12.82% and 5.85% of the *Myxococcales* identified in the EMP data, respectively, while none of the other 19 genera exceeded 3%. Notably, in almost all environments, at least 20% of the myxobacterial OTUs had not been classified at the family level, and at least 50% had not been classified at the genus level (Fig. S2). The unclassified myxobacterial OTUs were normally at high levels in environment types that had the lowest myxobacterial richness, i.e., the hypersaline and saline sediment and water.

The *Myxococcales* OTUs appeared in each of the 17 environment types but were very low in many environment types (Fig. 2b; detailed read number and proportion of different myxobacterial families and genera in the 17 environment types are summarized in Table S3). The highest relative abundance appeared in the plant rhizosphere samples (3.46%), followed by the nonsaline soil samples (2.23%) and then the nonsaline sediment samples (1.63%). The *Myxococcales* OTUs were rare in all animal-associated samples (<0.1% of the total OTUs). Notably, 9 out of 10 myxobacterial families were found in at least 15 environment types, and 16 out of 21 myxobacterial genera appeared in at least 10 environment types. The results indicated that many myxobacterial taxa are widely distributed in different environmental conditions. *Polyangiaceae*, Cystobacteraceae, and *Haliangiaceae* were the top 3 myxobacterial families, together accounting for more than 50% of the total myxobacterial OTUs. These three myxobacterial families had high richness in the nonsaline environments. In the hypersaline samples, only the families of *Kofleriaceae* and *Nannocystaceae* were detected. However, these two families, appeared in almost all 17 environment types except the animal corpus and proximal gut samples. The results suggested that *Kofleriaceae* and *Nannocystaceae* were highly resistant to various environmental conditions. In the plant rhizosphere, soil (nonsaline), and sediment (nonsaline) environments, the relative abundance of the *Polyangiaceae*, Cystobacteraceae, and *Haliangiaceae* families exceeded 0.2%, and *Myxococcaceae* had the highest relative abundance in the plant rhizosphere. In contrast, *Sandaracinaceae* had the highest relative abundance on the plant surface (0.10%). Thus, different myxobacterial families probably have preferred environments.

At the genus level, *Haliangium* of *Haliangiaceae*, *Cystobacter* of Cystobacteraceae, and *Chondromyces* of *Polyangiaceae* were the top 3 among the 21 myxobacterial genera in the environment (Fig. 2b). *Cystobacter* had a high relative abundance in not only the plant rhizosphere but also in nonsaline aerosols. According to the EMP data, the *Anaeromyxobacter* genus, which is a validly published facultative anaerobic myxobacterium (7), had a high relative abundance in the plant rhizosphere and nonsaline sediment. *Haliangium* is a typical representative of marine myxobacteria (37), and the presently cultured *Haliangium* strains contain two species, both of which were isolated from coastal saline environments (38). Our analysis results from the EMP data showed that *Haliangium* was not only predominant in saline environments, but also widely distributed in various other environments. Overall, the relative abundance of myxobacteria in the saline environments was lower than that in the nonsaline environments, suggesting that salinity was an important influencing factor.

### Cultured and genome-sequenced myxobacteria are mostly the highly abundant and widely distributed taxa.

**FIG 3:**
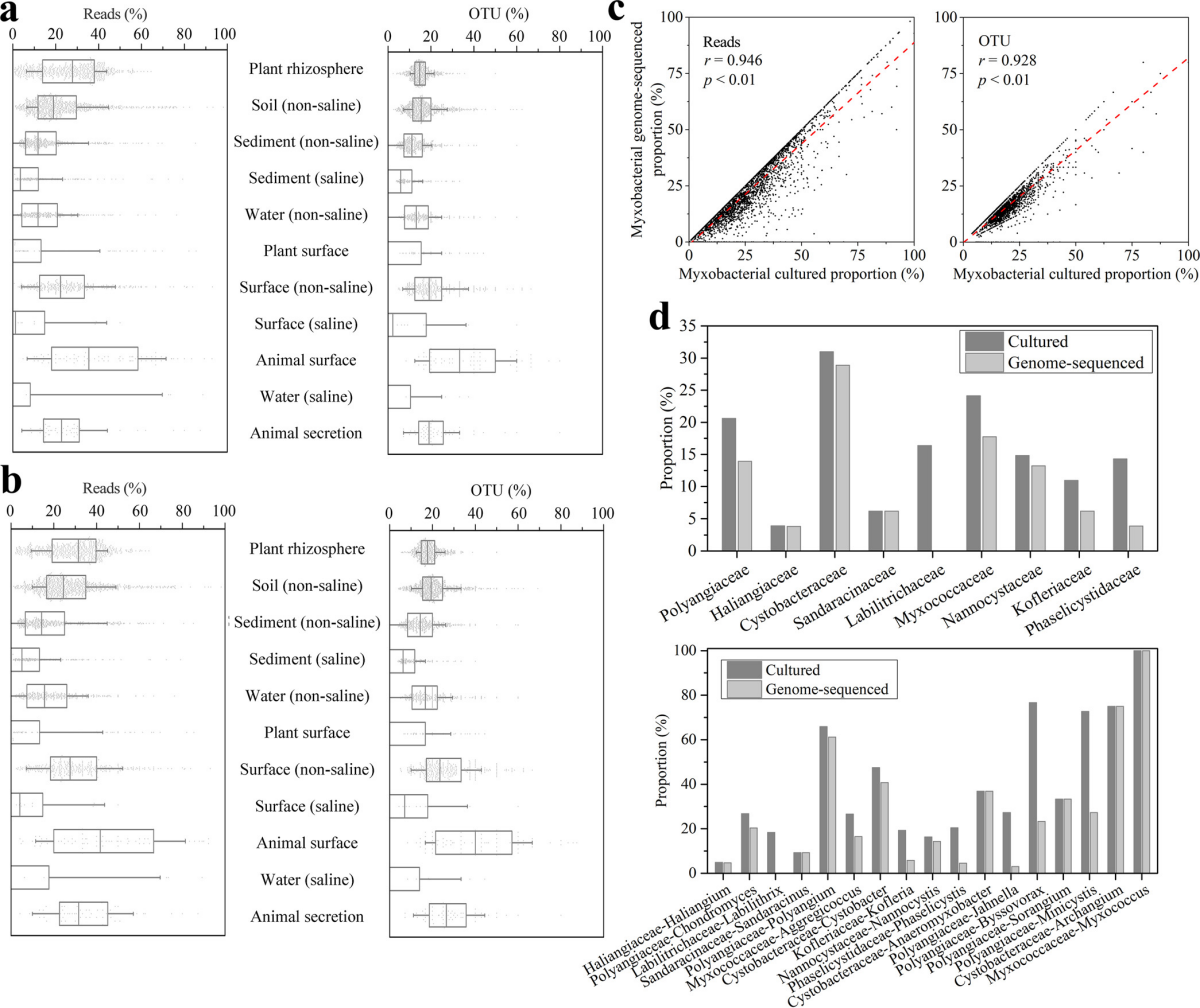
Proportions of cultured and genome-sequenced myxobacterial reads and taxa in different environment types containing at least 5 myxobacterial OTUs and at least 20 such samples. (a) Proportion of cultured myxobacterial reads and taxa. (b) Proportion of genome-sequenced myxobacterial reads and taxa. OTUs share 100% identities with the sequenced genomes. Each gray point represents a single sample. For the box plots, the middle line indicates the median, the box represents the 25th to 75th percentiles, and the error bar indicates the 10th to 90th percentiles of observations. (c) Correlation between cultured and genome-sequenced proportions of myxobacteria. (d) Cultured and genome-sequenced myxobacteria at the family and genus levels.

We collected the 16S rRNA gene sequences of cultured myxobacteria from the Ezbiocloud and Refseq databases and estimated their proportions among the myxobacteria revealed from the EMP data. Among the EMP myxobacterial OTUs, 614 OTUs were closely related to the cultured myxobacteria at the species level (at >97% identities of the 16S-V4 region), accounting for 10.01% of all the myxobacterial OTUs revealed from the EMP data. Fig. 3a shows the cultured myxobacterial read and OTU proportions in the samples of 11 environment types that contained at least 5 myxobacterial OTUs and at least 20 such samples (a total of 3,461 samples). The median cultured proportions at the species level were 20.31% (8.60% to 34.29%) and 16.92% (10.53% to 23.08%) of the myxobacterial reads and OTUs in different environments, respectively. That is, in half of the environmental samples, the reads and OTUs that are closely related to the cultured myxobacteria accounted for at least 20.31% of all myxobacterial reads and at least 16.92% of all myxobacterial OTUs, respectively.

In terms of the environment, the animal surface samples had the highest culture rate of myxobacteria, and the medians of the cultured read and OTU proportions both reached approximately 40%. The median proportions of cultured myxobacterial reads in the plant rhizosphere and nonsaline soil samples were 31.44% and 24.45%, respectively, both of which were significantly higher than the medians of cultured myxobacterial OTUs (17.65% and 19.35%). Comparably, the cultured median proportions of myxobacterial reads and OTUs were both less than 7% in the saline sediment, surface, or water samples, but were more than 14% in the nonsaline sediment, surface, and water samples. The results indicated that more myxobacteria have been cultured in nonsaline environments than in saline environments.

We further collected the genome-sequenced myxobacteria from the Refseq database to estimate the genome-sequenced proportion of myxobacteria. A total of 447 myxobacterial OTUs were genome-sequenced at the species level (at >97% identity of the 16S-V4 region), accounting for 7.29% of the total myxobacterial OTUs, which was lower than our recent estimation of the prokaryotes that have been genome sequenced (12.2%) (39). In the environment types containing at least 5 myxobacterial OTUs and at least 20 such samples (a total of 3,461 samples in 11 environment types), the median proportions of reads and OTUs that were closely related to genome-sequenced myxobacteria at the species level were 15.73% (6.25% to 29.17%) and 13.85% (8.33% to 19.05%), respectively (Fig. 3b). Similar to that of the cultured myxobacteria, the genome-sequenced myxobacteria in saline samples were much less than that in the nonsaline samples. Thus, at present, marine myxobacteria are greatly underrepresented.

The genome-sequenced proportions of myxobacterial reads and OTUs were both highly positively correlated with the cultured proportions in different samples (*r* = 0.946, *P* < 0.01; *r* = 0.928, *P* < 0.01, respectively) (Fig. 3c). Notably, among the 22 prokaryotic orders containing at least 1,000 OTUs, the genome-sequenced proportion of *Myxococcales* was the second lowest; it was only higher than *Plantomycetales* (4.40%). Among the 66 prokaryotic orders that contained 100 to 1,000 OTUs, the genome-sequenced proportion of *Myxococcales* was ranked 62nd. The results indicated that the genome information of the *Myxococcales* members was mostly unknown.

The cultured proportion also varied greatly between different myxobacterial taxa revealed from the EMP data (Fig. 3d). At the family level, Cystobacteraceae had the highest cultured proportion, reaching 30.99%, while the cultured proportions of *Polyangiaceae* and *Haliangiaceae* accounted for 20.60% and 3.92%, respectively. At the genus level, the cultured proportion was as high as 100% for *Myxococcus* but was only 4.8% for *Haliangium*, which contained the largest number of myxobacterial OTUs. The genome-sequenced proportion also varied greatly among the different myxobacterial taxa (Fig. 3d).

### Environmentally superior myxobacteria.

**FIG 4:**
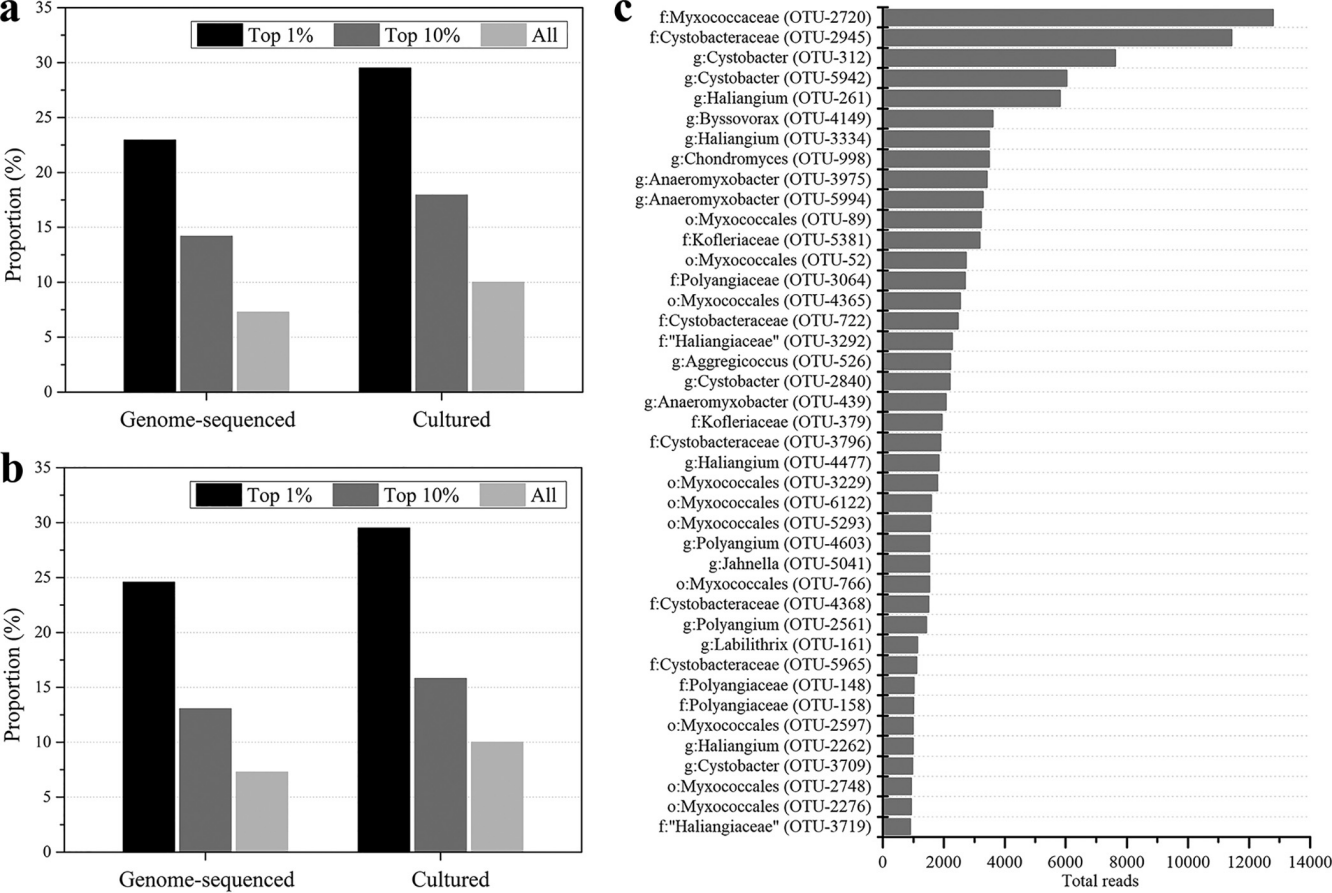
The environmentally superior myxobacterial taxa. (a) Correlations of the genome-sequenced and cultured proportions with the predominant myxobacterial taxa. (b) Correlations of the genome-sequenced and cultured proportions with the widespread myxobacterial taxa. Predominant taxa refer to the myxobacterial OTUs ranked in the top 1% or 10% in the read abundance. Widespread taxa refer to the myxobacterial OTUs ranked in the top 1% (existing in at least 250 samples) or 10% (existing in at least 40 samples) in the sample distribution. (c) The environmentally superior myxobacterial taxa and their global appearance (detailed information refers to Table S4 in the supplemental material).

It is known that a few top taxa make up the majority of read abundance (40). Consistently, our analysis also showed that the top 1% of myxobacterial OTUs accounted for 43.08% of the total myxobacterial read abundance, and the top 10% of myxobacterial OTUs accounted for 80.26%. The myxobacteria that have been cultured and genome sequenced accounted for 29.51% and 22.95% of the top 1% of myxobacterial OTUs, 17.94% and 14.19% of the top 10%, and 10.01% and 7.29% of all myxobacterial OTUs (Fig. 4a), respectively. Similarly, the myxobacteria with wide distributions were more likely to have been cultured and genome sequenced (Fig. 4b). The top 1% of myxobacteria that were most widely distributed (existing in at least 250 samples) made up proportions of 29.51% and 24.59% of the cultured and genome-sequenced myxobacteria, and the corresponding values for the top 10% of myxobacterial OTUs (present in at least 40 samples) were 15.82% and 13.05%, respectively.

The overlapping myxobacterial OTUs between the top 1% for abundance and the top 1% for wide distribution comprised a set of 41, and their relative abundance accounted for approximately two-thirds of the total. We refer to these 41 OTUs as the environmentally superior myxobacterial OTUs (Fig. 4c; details refer to Table S4). These environmentally superior OTUs come from 6 families and 9 genera, but their proportions were significantly different at the family and genus levels. The environmentally superior OTUs from Cystobacteraceae were the most abundant, accounting for 29.27% of all the environmentally superior OTUs of myxobacteria OTUs. *Cystobacter* and *Anaeromyxobacter* of Cystobacteraceae accounted for 9.76% and 7.32% of the environmentally superior OTUs, respectively. Notably, more than 60% of these superior myxobacteria OTUs had not been cultured, and more than 70% had not yet had their genomes sequenced.

## DISCUSSION

For a long time, myxobacteria have been considered terrestrial bacteria. Accordingly, a large number of early isolations of myxobacteria focused on the soil environment. For example, Reichenbach and his group were devoted to the isolation of myxobacterial resources from various terrestrial environments and collected more than 10,000 myxobacterial strains over several decades (3, 14). Wolfgang Dawid also doggedly isolated myxobacterial resources from more than a 1,000 soil samples collected in 64 countries on all continents (13). Accordingly, the vast majority of characterized myxobacteria were obtained from terrestrial habitats prior to 2000 (37). During the past 20 years, an increasing number of myxobacteria have been isolated using classical or modified isolation methods for the discovery of new myxobacteria. For example, many halophilic myxobacteria, in addition to halotolerant myxobacteria (15), have been isolated from marine environments; these species have included Plesiocystis pacifica, Enhygromyxa salina, Pseudenhygromyxa salsuginis, and Haliangium ochraceum, and their characteristics are different from those of terrestrial myxobacteria (38, 41–46). These marine myxobacteria resources are considered an excellent candidate source of secondary metabolites with unique chemical scaffolds (47). In addition, Garcia et al. reaffirmed the distinctions among the three suborders of myxobacteria, discovered nine new taxa, and expanded the phylogeny of myxobacteria (48). Through a systematic metabolite survey of approximately 2,300 myxobacterial strains, Hoffmann et al. found a positive correlation between taxonomic distance and the production of distinct secondary metabolites, which further supports the idea that the chances of discovering novel metabolites are greater when examining strains from new genera (4). In rhizosphere, Cystobacteraceae and *Polyangiaceae* were found in the biomarker list revealed in root microbiota, probably important for the differentiation of *indica* and *japonica* (49). Similarly, the OTU number of myxobacteria accounted for 4.0% (130/3211) in rhizosphere and bulk soil in maize roots (50). Diverse and widespread myxobacteria across heterogeneous environments on a global scale probably play important ecological functions, and determination of the roles of myxobacteria requires isolation of them. However, the present isolation of myxobacterial resources is normally random with no guide for myxobacterial distribution.

This study displayed a panoramic view of the myxobacteria on Earth. Through the comparative analysis of the EMP data and public information, we determined that *Myxococcales* is one of the most diverse known prokaryotic orders on Earth, especially in soil, sediments, and freshwater environments, which was consistent with previous extensive explorations (20, 22–26, 33). A recent study identified 511 dominant phylotypes as the most abundant and ubiquitous bacteria (accounting for 2% of the total) in soils from across the globe (40). We found that 11 myxobacterial OTUs were among the dominant, which were assigned to *Polyangiaceae* (4 OTUs), *Haliangiaceae* (3 OTUs), *Myxococcaceae* (2 OTUs), and Cystobacteraceae (2 OTUs). Consistently, the globally environmentally superior myxobacterial OTUs revealed in our study also include these three families. In addition to the cultured groups, there are still a large number of unclassified myxobacteria in almost all environments. Compared with the recently estimated 52.0% ± 24% of sequences (number of reads) and 34.9% ± 23% of taxa for closely related cultured relatives in the prokaryotes (51), myxobacteria are among the least cultured groups. Analogously, the rate of sequenced myxobacterial genomes is also lower than that recently estimated for the prokaryotes (7.29% versus 12.2%) (39). At present, the cultured and genome-sequenced proportions of myxobacteria vary greatly among different environment types and different taxa, and myxobacteria are still one of the least commonly cultured and genome-sequenced prokaryotes. The global geographic distribution of the myxobacterial community facilitates our understanding of the diversity and ecology of myxobacteria and helps in the development of effective enrichment and isolation techniques for different myxobacterial resources for further application.

The global survey presented in this paper indicated that myxobacteria rarely appeared in various host-associated environments. From their taxonomic information (Table S1), the myxobacteria revealed in host-associated environments were rather the same as those appearing in free-living environments. Based on the EMP data, it is difficult to analyze whether the host-associated myxobacteria are physiologically different from those in free-living environments. It is well known that most of the cultured myxobacteria are aerobic, and the only cultured facultative anaerobic myxobacterial taxon (*Anaeromyxobacter*) is from free-living conditions (7). According to the characteristics of cultured myxobacteria, we speculate that the myxobacterial sequences detected in host-associated environments are probably from the highly resistant fruiting bodies or myxospores of surrounding environments. However, there might be some host-associated myxobacterial taxa with specific traits, for example, Pajaroellobacter abortibovis, which has yet to be successfully cultured *in vitro* (52), and its specific physiological characteristics and living patterns remain to be further investigated.

Notably, the most easily obtained myxobacterial taxa, such as *Myxococcus*, *Corallococcus*, *Archangium*, and *Cystobacter*, have been cultured using the classical isolation method. *Myxococcus* and *Corallococcus* are also the most genome-sequenced myxobacteria. However, the global analysis showed that *Myxococcus* only accounted for 0.07% of the myxobacterial community, and sequences of *Corallococcus* were not detected in the EMP data analysis. This phenomenon indicated not only the bias of the classical isolation techniques for myxobacteria, but also the limitation of the EMP data. We used specific primers to enrich the 16S rRNA gene sequences of myxobacteria, which revealed that the myxobacteria were much more diverse in soil, oceanic sediments, or lake sediments (20, 22, 23, 25, 26). Thus, although we provided a global view of myxobacteria distribution and diversity, detailed information on myxobacterial distribution and diversity beyond the EMP data, mainly those rare myxobacteria, requires deeper sequencing and mining, such as using myxobacterium-specific techniques.

In conclusion, this study displays a panoramic view of myxobacteria. The myxobacteria are one of the most diverse bacteria on Earth. Different *Myxococcales* members have their preferred living environments, especially in soil, sediments, and freshwater environments. However, myxobacteria are among the least investigated bacterial groups. The rates of cultured and genome-sequenced myxobacteria varied greatly between different environment types and different families and genera. This study exhibits the global geographic distribution of the myxobacterial community and facilitates our understanding of the diversity and ecology of myxobacteria.

## MATERIALS AND METHODS

### Data collection from EMP.

The 16S rRNA gene fragments were obtained from the EMP, which employs a unified standard workflow for soil collection across the globe, metadata curation, and analysis to provide a robust interpretation of ecological trends across diverse environments on Earth (1, 34, 35). Sample processing, sequencing, and core amplicon data analysis were performed by the EMP (www.earthmicrobiome.org). The 16S rRNA gene sequence data from 10,000 samples have removed errors and trimming using Deblur software by the EMP (53). A total of 262,011 OTUs and their abundance and nucleic acid sequence information were collected from the website (http://ftp.microbio.me/emp/release1).

### Taxonomic and environmental analysis of EMP OTUs.

The taxonomy of each OTU was identified by the Ribosomal Database Project (RDP) classifier at a 70% confidence threshold (36). The *Myxococcales* sequences were selected for further analysis. The EMP Ontology (EMPO) classified the environments as free-living or host-associated (level 1), saline or nonsaline (if free-living) or animal or plant (if host-associated) (level 2), and described them using 17 environmental types (level 3) (1). Based on the taxonomic results and the EMPO (level 3) for each OTU, we calculated the proportion of myxobacterial reads and taxa in the prokaryotic biomes and the composition and relative abundance of myxobacteria at the family and genus levels in different environments.

### Estimate of the proportion of cultured and genome-sequenced myxobacteria.

The 16S rRNA gene sequences of the cultured myxobacterial type strains were obtained from the EzBioCloud and GenBank databases. The NCBI reference sequence (RefSeq) database is a curated nonredundant collection of complete or incomplete genome sequences (54). The 16S rRNA gene sequences of all genome-sequenced prokaryotes were obtained from the RefSeq database. The alignment between the EMP OTUs and the cultured or genome-sequenced myxobacterial 16S rRNA gene sequences was performed using BLASTn (at >97% identities and E value < 1e−5) (55). The cultured and genome-sequenced proportions of myxobacteria were calculated at the family and genus levels. We evaluated the cultured and genome-sequenced proportions of myxobacterial reads and taxa in different environments that contained at least 5 myxobacterial OTUs (a total of 3,461 samples). The OTUs that ranked in the top 1% for read abundance were defined as predominant myxobacterial taxa, and the OTUs that ranked in the top 1% for sample distribution were defined as widespread myxobacterial taxa. We referred to the overlapping myxobacterial OTUs between the predominant taxa and the widespread taxa as the environmentally superior myxobacterial OTUs. The cultured and genome-sequenced proportions of the predominant taxa, widespread taxa, and environmentally superiority OTUs were calculated.
