# Supplementary material for: Global Geographic Diversity and Distribution of the Myxobacteria
Source: Microbiol Spectr. 2021 Jul 14;9(1):10.1128/spectrum.00012-21. doi: 10.1128/spectrum.00012-21 (PMC8552515; doi:10.1128/spectrum.00012-21)
Supplement: SUPPLEMENTAL FILE 1 — Download SPECTRUM00012-21_Supp_1_seq1.pdf, PDF file, 0.4 MB [file spectrum00012-21_supp_1_seq1.pdf]

Supplemental material

Global geographic diversity and distribution of the myxobacteria

Jingjing Wang†[1], Jianing Wang†[1], Shuge Wu[1], Zheng Zhang*[1,2], Yuezhong Li*[1]

[1]State Key Laboratory of Microbial Technology, Institute of Microbial Technology, Shandong University, Qingdao 266237, P. R. China

[2]Suzhou Research Institute, Shandong University, Suzhou 215123, P. R. China

Supplemental legends

Fig. S1 The relative abundance of top 30 orders among the prokaryotic revealed from the EMP data at the global scale.

Fig. S2 Unclassified myxobacteria in different environment types.

Table S1. The myxobacterial OTUs identified from a 10,000-sample subset of the EMP data by the Ribosomal Database Project (RDP) classifier. 5,000 sequences were randomly selected in each sample for the calculation. The confidence threshold was set at the default value of 70%.

Table S2. Proportion and rank of myxobacterial OTUs in the 17 environment types.

Table S3. Read numbers and proportions of different families and genera of *Myxococcales* in the 17 environment types.

Table S4. Environmentally superior myxobacterial OTUs.

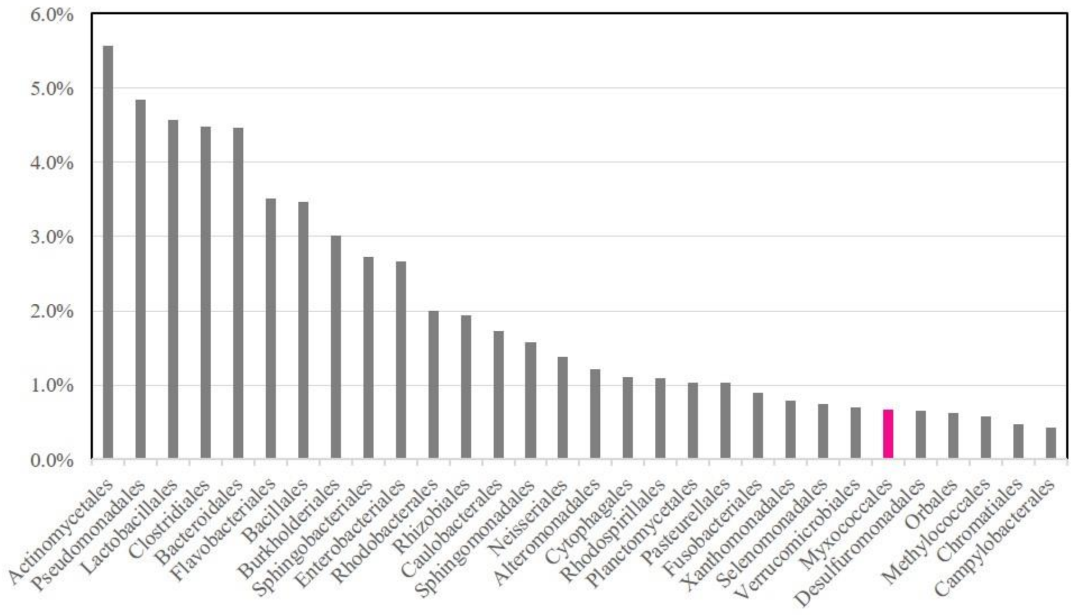

Fig. S1 The relative abundance of top 30 orders among the prokaryotic revealed from the EMP data at the global scale.

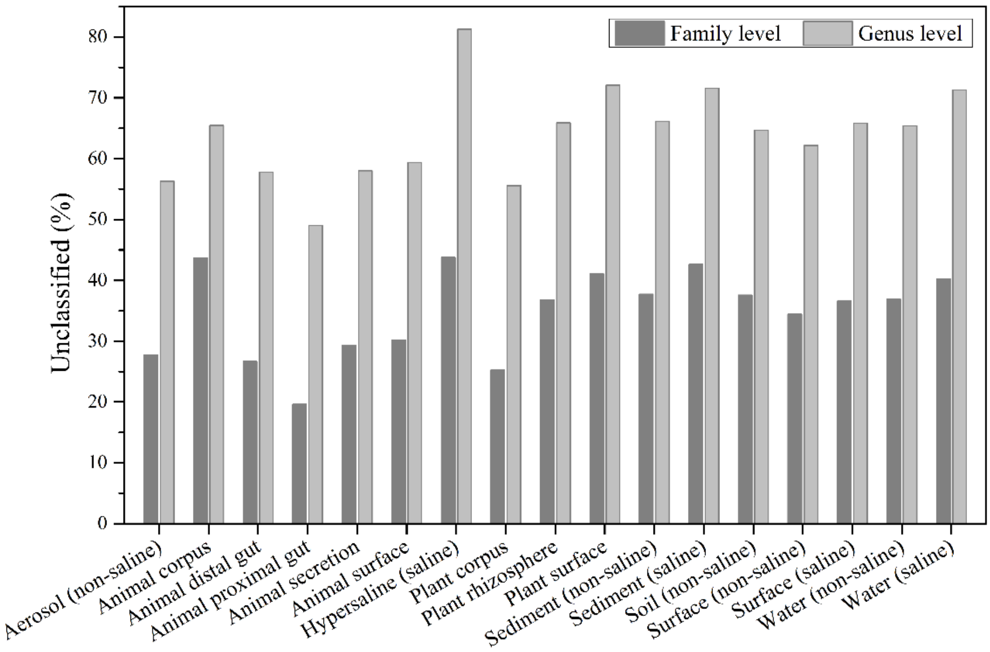

Fig. S2 Unclassified myxobacteria in different environment types.

Table S2. Proportion and rank of myxobacterial OTUs in the 17 environment types.

EMPO	Samples	Total OTUs	Myxo OTUs	Myxo proportion	Ranking among 144 orders
Sediment (non-saline)	544	50989	1737	3.41%	1
Soil (non-saline)	954	93287	3627	3.89%	2
Plant rhizosphere	552	39483	1471	3.73%	2
Water (non-saline)	954	77551	1804	2.33%	2
Sediment (saline)	541	45830	777	1.70%	4
Surface (non-saline)	953	44178	1422	3.22%	4
Animal secretion	917	19302	610	3.16%	5
Plant surface	953	13839	358	2.59%	5
Surface (saline)	117	17425	246	1.41%	9
Animal surface	987	31655	652	2.06%	10
Water (saline)	682	17501	216	1.23%	10
Plant corpus	123	4201	99	2.36%	11
Hypersaline (saline)	13	2118	16	0.76%	14
Aerosol (non-saline)	81	10129	119	1.17%	15
Animal corpus	322	3603	55	1.53%	15
Animal distal gut	953	26697	225	0.84%	15
Animal proximal gut	354	7667	51	0.67%	21
All	10000	262011	6133	2.34%	4
